# Small compound 6-*O*-angeloylplenolin induces caspase-dependent apoptosis in human multiple myeloma cells

**DOI:** 10.3892/ol.2013.1370

**Published:** 2013-05-30

**Authors:** YING LIU, YING DONG, BO ZHANG, YONG-XIAN CHENG

**Affiliations:** 1 The Shenzhen Key Lab of Gene and Antibody Therapy, Center for Biotech and BioMedicine and Division of Life Sciences, Shenzhen Graduate School, Tsinghua University, Shenzhen, Guangdong 518055, P.R. China; 2 Department of Oncology, The Second Affiliated Hospital, College of Medicine, Zhejiang University, Hangzhou, Zhejiang 310009, P.R. China; 3 National Laboratory of Biomacromolecules, Institute of Biophysics, Chinese Academy of Sciences, Beijing 100101, P.R. China; 4 State Key Laboratory of Phytochemistry and Plant Resources in West China, Kunming Institute of Botany, Chinese Academy of Sciences, Kunming, Yunnan 650204, P.R. China

**Keywords:** 6-*O*-angeloylplenolin, multiple myeloma, apoptosis, caspase

## Abstract

6-*O*-angeloylplenolin (6-OAP) is a sesquiterpene lactone agent that has been previously demonstrated to inhibit the growth of multiple myeloma (MM) cells through mitotic arrest with accumulated cyclin B1. In the present study, the levels of apoptosis were analyzed in dexamethasone-sensitive (MM.1S), dexamethasone-resistant (U266) and chemotherapy-sensitive (RPMI 8226) myeloma cell lines. Enhanced apoptosis was identified following a 48-h incubation with 6-OAP (0–10 *μ*M) that induced a dose-dependent decrease in pro-casp-3 and the cleavage of its substrate, anti-poly (ADP-ribose) polymerase (PARP). In addition, time-dependent cleavage of PARP was also detected in U266 and MM.1S cells. The mechanism of 6-OAP cytotoxicity in all cell lines was associated with the induction of apoptosis with the presence of cleaved caspase-3 and PARP. In conclusion, 6-OAP-induced apoptosis is caspase-dependent. These observations are likely to provide a framework for future studies of 6-OAP therapy in MM.

## Introduction

Multiple myeloma (MM) or plasma cell myeloma is a malignant disorder characterized by the accumulation of differentiated B cells (plasma cells). The incompletely differentiated plasma cells are characterized by deregulated apoptosis (1). The treatment of MM remains unsatisfactory and new agents that specifically target key signaling pathways required for myeloma growth or survival are urgently required.

**Figure 1.**
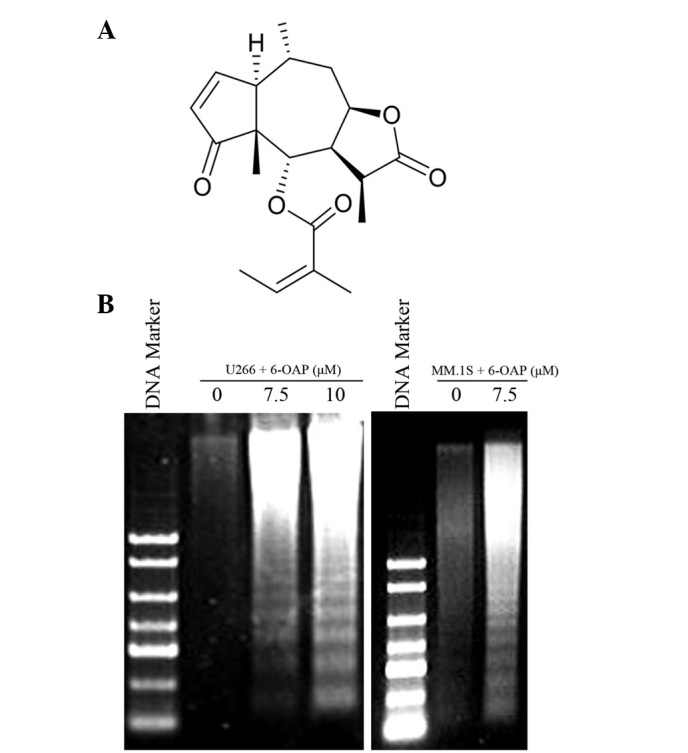
6-OAP induces apoptosis in multiple myeloma (MM) cells, as detected by DNA fragmentation assay. (A) Chemical structure of 6-OAP. (B) U266 and MM.1S cells were treated with 6-OAP at the indicated concentrations for 48 h and DNA fragmentation was analyzed. 6-OAP, 6-*O*-angeloylplenolin.

6-*O*-angeloylplenolin (6-OAP; Fig 1A) is a sesquiterpene lactone isolated from *Centipeda minima* that has been studied in hematological and solid forms of cancer and has been revealed to exhibit activity without significant toxicity (2–4). The results of our preliminary study demonstrated that 6-OAP inhibits the proliferation of human MM cells by inducing the arrest of mitosis and inhibiting specific key pathways (5). However, whether 6-OAP-induced mitosis arrest and pathway inhibition are followed by apoptosis requires further study. Therefore, the aim of the present study was to investigate the apoptotic effect of 6-OAP against human myeloma cells.

## Materials and methods

### Reagents

6-OAP with a purity ≤99.5% was extracted from *Centipeda minima* (L.) as described previously (4). The 6-OAP was then dissolved in DMSO (Sigma-Aldrich, St. Louis, MO, USA) to produce a stock solution of 10^−2^ M, which was stored at −20°C.

### Cell culture

MM.1S, U266 and RPMI 8226 human MM cell lines were purchased from the American Type Culture Collection (Manassas, VA, USA). The cells were cultured in RPMI-1640 medium supplemented with 10% (for U266) or 15% (for RPMI 8226 and MM.1S) fetal bovine serum (Hyclone Laboratories, Inc., Logan, UT, USA) and incubated in a humidified atmosphere with 5% CO_2_ at 37°C.

### Patient samples

CD138^+^ cells from a single patient with MM were isolated with informed consent from bone marrow (BM) mononuclear cells using positive immunomagnetic column separation (Miltenyi Biotec GmbH, Bergisch Gladbach, Germany). The purity of the CD138^+^ cells was >97% as determined by flow cytometry. This study was approved by the ethics committee of Shenzhen Graduate School, Tsinghua University, Shenzhen, China.

### DNA fragmentation

The MM cells were collected and lysed in 0.5 ml lysis buffer containing 10 mM Tris (pH 8.0), 10 mM EDTA and 0.05% Triton X-100. The lysate was centrifuged, RNase (0.2 mg/ml) was added and the lysate was incubated for 30 min at 37°C. Proteinase K (0.1 mg/ml) and sodium dodecyl sulfate (SDS; final concentration 1%) were added, followed by incubation at 50°C for 16 h. DNA was extracted with phenol/chloroform and then chloroform, prior to being precipitated with ethanol and sodium acetate and electrophoresed on 1.5% agarose gels, and then visualized with ethidium bromide (EB) staining.

### Flow cytometric assays for Annexin-V (AV)

Cell apoptosis was evaluated by AV detection using an AV-FITC kit (BD Biosciences, Franklin Lakes, NJ, USA), according to the manufacturer’s instructions.

### Western blot

Cell pellets were lysed in RIPA buffer containing 50 mM Tris (pH 8.0), 150 mM NaCl, 0.1% SDS, 0.5% deoxycholate, 1% NP-40, 1 mM DTT, 1 mM NaF, 1 mM sodium vanadate and a protease inhibitor cocktail (Sigma-Aldrich). Protein extracts were quantitated, loaded on 8–12% SDS-polyacrylamide gels, electrophoresed and then transferred to a nitrocellulose membrane (Whatman plc, Maidstone, Kent). The membrane was incubated with primary antibody, washed and incubated with horseradish peroxidase-conjugated secondary antibody. Detection was performed using a chemiluminescent western detection kit (Cell Signaling Technology, Inc., Danvers, MA, USA). The antibodies used were anti-caspase-3, anti-poly (ADP-ribose) polymerase (PARP; Cell Signaling Technology, Inc.) and anti-β-actin (Santa Cruz Biotechnology, Inc., Santa Cruz, CA, USA).

### Statistical analysis

All experiments were repeated at least three times and the data are presented as the mean ± SD unless noted otherwise. P<0.05 was considered to indicate a statistically significant difference.

## Results

### 6-OAP induces apoptosis in MM cells

The levels of apoptosis were analyzed using the DNA fragmentation assay in dexamethasone-sensitive (MM.1S) and dexamethasone-resistant (U266) myeloma cell lines treated with 6-OAP. As demonstrated in Fig. 1B, marked DNA ladders were observed in MM.1S and U266 cells treated with 6-OAP, indicative of apoptosis detection.

**Figure 2.**
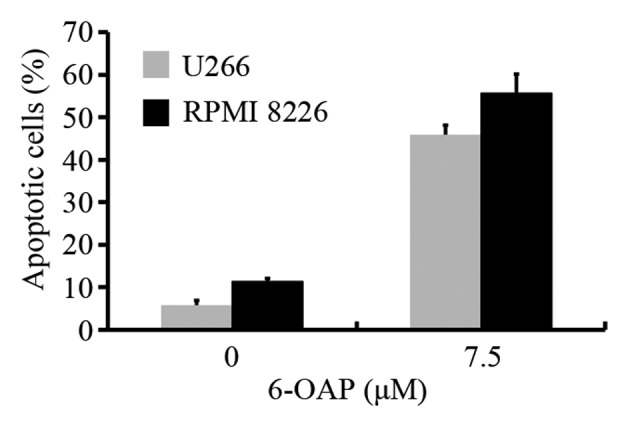
6-OAP induces apoptosis in multiple myeloma (MM) cells detected by Annexin V staining. U266 and RPMI 8226 cells were treated with 6-OAP for 24 h. Annexin V staining was determined by flow cytometry. 6-OAP, 6-*O*-angeloylplenolin.

In addition, AV staining was conducted to assess apoptosis in U266 and chemotherapy-sensitive RPMI 8226 cell lines treated with 6-OAP. Using flow cytometry, 7.5 *μ*M 6-OAP was identified to induce apoptosis at a ratio of 28 and 46% in U266 and RPMI 8226 cells, respectively (Fig. 2). These results indicate that 6-OAP induces apoptosis in MM cells.

### 6-OAP-induced apoptosis in MM cells is caspase-dependent

**Figure 3.**
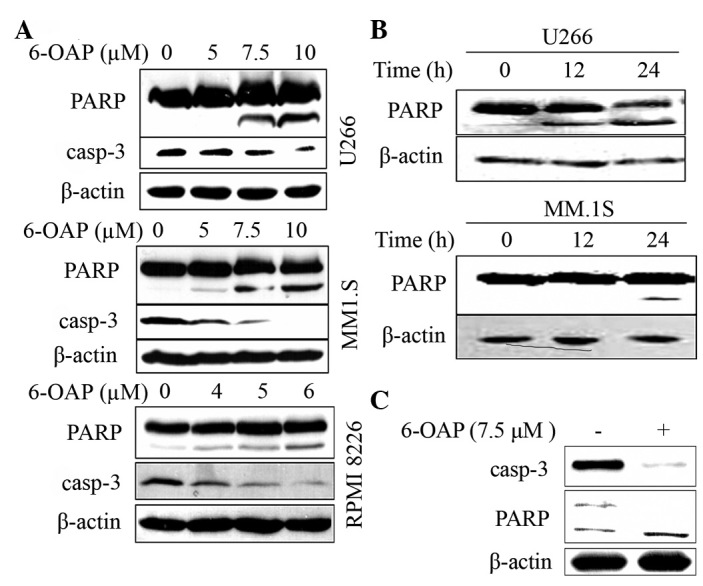
6-OAP induces caspase-dependent apoptosis of multiple myeloma (MM) cells. (A) U266, MM.1S and RPMI 8226 cells were treated with 6-OAP for 48 h and lyzed, then extracts were subjected to western blot analysis. (B) U266 cells (upper) or MM.1S cells (lower) were treated with 6-OAP for the indicated time points. (C) CD138^+^ primary cells isolated from 1 MM patient were treated with or without 6-OAP for 24 h. Total cell lysates were subjected to western blot analysis using antibodies against casp-3, PARP and β-actin. 6-OAP, 6-*O*-angeloylplenolin; casp-3, caspase-3; PARP, anti-poly (ADP-ribose) polymerase.

The apoptotic pathways that ultimately lead to the activation of effector caspases (casp-3, -2 and -7) and the cleavage of PARP have been characterized in MM (6). Therefore, a western blot analysis was used to detect the activation of the casp-3 effector caspase and its substrate, PARP, in the MM cells. 6-OAP was demonstrated to induce a significant dose-dependent decrease in pro-casp-3 and the cleavage of its substrate, PARP, in the three cell lines, indicating the activation of casp-3 (Fig. 3A). 6-OAP also markedly induced the cleavage of PARP in a time-dependent manner in the U266 and MM.1S cells (Fig. 3B). In addition, the expression of pro-casp-3 and the cleavage of PARP was investigated in CD138^+^ primary cells isolated from a single MM patient (Fig. 3C). The results of the western blot analysis demonstrated that 6-OAP significantly induces the activation of casp-3. These observations indicate that 6-OAP induces caspase-dependent apoptosis in MM cells.

## Discussion

The natural agent, 6-OAP, was initially demonstrated to exhibit anti-bacterial and anti-protozoal activities (7–9). However, more recently, studies have demonstrated an anti-tumor activity for 6-OAP in solid tumors and hematological malignancies (2–4). Our previous observations found that 6-OAP induces the arrest of mitosis in MM cells by the activation of the spindle assembly checkpoint and the accumulation of cyclin B1. In addition, 6-OAP was identified to inhibit the Jak2/Stat3 and Akt signaling pathways, thereby blocking the facilitation of the BM microenvironment on the MM cells. 6-OAP has also been found to induce marked inhibition of NF-κB in MM cells (5). However, to date, no studies have determined whether the inhibitory effects of 6-OAP on the cell cycle and certain signal pathways ultimately result in apoptosis. Therefore, in the present study, the effect of 6-OAP on apoptosis in MM cells was analyzed.

Apoptosis is an active process that ultimately leads to the activation of endonucleases and the cleavage of DNA into fragments of 180–200 bp. The extrinsic and intrinsic apoptotic pathways that ultimately lead to activation of effector caspases (casp-3, -2 and -7) have also been characterized (10,11). The present study demonstrated that 6-OAP-treated MM cells exhibited evident DNA fragments of 180–200 bp (Fig. 1B), indicating that 6-OAP induces apoptosis in MM cells. The activation of the effector caspases was also analyzed and 6-OAP was found to induce casp-3 activation, followed by PARP cleavage in various MM cell lines (Fig. 3), indicating that 6-OAP induces caspase-dependent apoptosis in MM cells. In conclusion, 6-OAP induces growth inhibition in human MM cells using a number of different mechanisms, including the arrest of mitosis and the inhibition of certain signaling pathways. These different mechanisms ultimately lead to caspase-dependent apoptotic cell death.
